# Prevalence and molecular epidemiology of CMV and EBV among nurses working in pediatrics, transplantology, and primary health care

**DOI:** 10.1002/1348-9585.12112

**Published:** 2020-02-07

**Authors:** Patrycja W. Zając, Bożena Czarkowska‐Pączek, Aleksandra Wyczałkowska‐Tomasik

**Affiliations:** ^1^ Department of Clinical Nursing Faculty of Health Sciences Medical University of Warsaw Warsaw Poland; ^2^ Department of Immunology Transplantology, and Internal Diseases Medical University of Warsaw Warsaw Poland

**Keywords:** cytomegalovirus, Epstein‐Barr virus, nurses, occupational health, job safety, exposure risk

## Abstract

**Objective:**

The aim of the study was to determine and compare the prevalence of cytomegalovirus (CMV) and Epstein‐Barr virus (EBV) antibodies and DNA among nurses working in different profiles of healthcare activity.

**Material and methods:**

The study population comprised 120 women (90 exposed healthcare workers and 30 controls). Blood samples were investigated using chemiluminescent microparticle immunoassays (CMIA) tests to detect the presence of EBV VCA IgM, IgG, and CMV IgM, IgG. Plasma CMV and EBV DNA levels were assessed using real‐time polymerase chain reaction (PCR).

**Results:**

CMV IgG antibodies were present in 87.80% nurses (86.70% in controls), EBV IgG were present in all the nurses studied and in the control group. No statistically significant differences were noted between the subgroups of nurses and the control group as regards IgG CMV, VCA IgG EBV. CMV IgM/EBV IgM antibodies were negative in all the nurses. CMV/EBV DNA was reported only in the study group. It was not found in any of control group participants.

**Conclusions:**

The positive PCR CMV/EBV markers only in the study group can be indicative of the exposure of nurses to these pathogens being greater than in other people not being professionally involved in patient care. In addition, it was observed that the level of CMV IgG antibodies as well as EBV VCA IgG antibodies tended to be linked to the age and the length of work of nurses working in pediatrics.

## 1 INTRODUCTION

Work in health care entails exposure to factors (chemical, physical, psychosocial, biological) commonly present in health care in the process of performing therapeutic, nursing or diagnostic services.^1^ Harmful biological factors constitute one of the essential components of the working environment which can cause viruses, bacteria, and fungi induced infectious and invasive diseases in employees.^2^ The scope and character of the work of the nurse generates a risk of exposure to harmful biological factors and consequently a risk of CMV as well as EBV transmission due to, first of all, direct contact with the patient, the patient's blood, body fluid, secretions, and excretions.^3^ What can further significantly contribute to disturbances in the function of the immunological system^4^ and thus to the increased susceptibility of nurses to infections is also the disturbance of the circadian rhythm, exposure to permanent stress, tension, work overload. The transmission of infection with CMV/EBV in the course of performing nursing and diagnostic tasks which constitute an element inherent to the nurse's work cannot thus be excluded. In particular, since it has repeatedly been pointed out that employees of healthcare entities seem to have difficulties in observing proper hand hygiene.^5^ And it is the observance of proper prophylactic practices, the use of gloves, hand washing, and disinfection, that tend to be recognized ways of preventing CMV and EBV infections.^6^ That is why routine diagnostics of healthcare workers toward CMV and EBV infections is not conducted these days. On the other hand, this may also be a source of decreased awareness of the always present threat resulting from exposure to these pathogens in the workplace. Consequently, what still remains to be answered is the question of whether in the course of performing professional duties the professional group of nurses in Poland are actually exposed to CMV and EBV transmission to a higher extent than the population having no professional links with health care.

The aim of the study was to determine and compare the prevalence of CMV and EBV specific antibodies and DNA among nurses working in different profiles of healthcare activity and in the control group of people not working in healthcare entities.

## 2 MATERIAL AND METHODS

The study group included 90 volunteers, professionally active nurses aged 24‐65 years (mean age 49.30), recruited from hospitals and a family medicine outpatient clinic in Warsaw, Poland. Because of the hypothesis of an increased risk in certain occupational populations, the sampling of nurses was not accidental. The areas of health care selected for the study covered nurses specializing in the care of patients exposed to a high risk of CMV/EBV infections and working in closer contact with patients (ie, in pediatrics, transplantology) than other workers those who were likely to be at a lower risk of CMV infection (primary health care). The study group was divided into three subgroups by place of work (n = 30 in each subgroup): First subgroup—pediatrics, Second subgroup—transplantology, Third subgroup—primary health care.

The control group included 30 women aged 24‐65 (mean age 47.90), volunteers, not working in health care and not working in direct contact with children.

All participants completed a self‐administrative questionnaire designed to collect demographic and occupational information.

The study was approved by the Bioethics Committee of the Medical University of Warsaw (No. KB/140/2014). A signed personal permission was also required from each participant.

### 2.1 Blood collection

A total of 120 blood samples were collected from March to April 2015. Venous blood (14 mL) was collected using a closed vacuum system BD Vacutainer® (Becton Dickinson, Poland). Samples were placed in two test tubes, for the serum analysis (10 mL) with a coagulation activator and for the plasma analysis (4 mL) with anticoagulant (K2‐EDTA). At the specialist laboratory of the Clinic of Immunology, Transplantology and Internal Diseases at The Infant Jesus Teaching Hospital in Warsaw (Poland) the specimens were centrifuged for 2 hours after the collection and stored at −80°C until the analysis was done. Each test was repeated once.

### 2.2 Laboratory method

#### 2.2.1 CMIA

Measurement of the serum levels of antibodies against CMV (IgM, IgG) and EBV (IgM, IgG) was performed by the CMIA with the use of the test for CMV and EBV (Abbott Laboratories) using the ARCHITECT and 1000 Abbott (Abbott Laboratories). Serologic tests were performed according to the manufacturer's instructions. Before performing the measurement of the concentration of antibodies in the serum a calibration test was done, ARCHITECT IgM or IgG Calibrator (Abbott Laboratories), and a quality control test, ARCHITECT IgM or IgG Controls, for the corresponding virus (Abbott Laboratories). Serum was thawed at room temperature and then stirred, in accordance with the test procedure, before the measurement, to obtain a homogeneous sample. The results were obtained through the automatic calculation by an analyzer.

Samples were considered as CMV IgG positive with concentration values ≥6.00 AU/mL and values ≥ 1.00 S/CO for CMV IgM antibodies. Samples were considered as EBV VCA IgG/IgM, positive with concentration values ≥ 1.00 S/CO, as recommended by the manufacturer.

### 2.3 Isolation of DNA

Isolation of DNA was performed in BioRobot EZ1 (Qiagen) using EZ1 Virus Mini Kit v2.0 (Qiagen) according to the manufacturer's instruction. After the quality and quantity control with the ND‐1000 camera (NanoDrop Technologies). DNA was stored at −80°C.

### 2.4 Real‐Time PCR

Real‐Time PCR reaction was performed using ABI 7500 (Applied Biosystems). Reaction mixture was successively added to each well of a MicroAmp Optical 96‐well reaction plate containing a barcode (Applied Biosystems). The reaction mixture included: TaqMan Universal PCR Master Mix (Applied Biosystems) and appropriately selected primers TaqMan Gene Expression Assay (Applied Biosystems) for EBV gene. CMV DNA was determined using CMV PCR Kit by GeneProof (GeneProof) according to the manufacturer's instruction. Gene expression levels were calculated using a standard curve.

### 2.5 Statistical analysis

The statistical analyses were carried out using: Statistica 10 and IBM SPSS Statistics 19. The differences between groups were analyzed using the Chi‐square or the Kruskal‐Wallis Test or the Mann‐Whitney U Test, as appropriate. The correlation was analyzed using the Sperman's rank correlation coefficient. A *P*‐value of < .05 was considered as statistically significant.

## 3 RESULTS

The study included a total of 120 blood samples from 90 nurses (exposed healthcare workers) and 30 women with an administrative job as a control group. The sample of nurses from primary health care differed significantly in *the age* and *the length of work* from nurses working in pediatrics, transplantology, and from the control group. (Table 1).

**Table 1:** Mean age and length of work in study subgroups and control group

	(1) Nurses pediatrics (n = 30)	(2) Nurses transplantology (n = 30)	(3) Nurses primary health care (n = 30)	(4) Control group (n = 30)
Age (y)				
Mean ± SD	48.53 ± 10.37	43.67 ± 8.50	55.57 ± 7.83^a^, *	47.97 ± 11.39
Range	24‐65	27‐60	28‐65	26‐65
Length of work (y)				
Mean ± SD	20.93 ± 13.30	19.87 ± 9.80	32.97 ± 8.66^b^, *	20.27 ± 10.41
Range	1‐44	3‐38	5‐46	2‐37

Abbreviation: SD, Standard Deviation.

a Kruskal‐Wallis Test: H = 22.81, *P* = .00**, Significant differences: 1/3, 2/3, 3/4

b Kruskal‐Wallis Test: H = 27.00, *P* = .00**, Significant differences: 1/3, 2/3, 3/4

### 3.1 Serology

#### 3.1.1 CMV

CMV IgG antibodies were detected in 79/90 (87.80%) nurses and 26/30 (86.70%) controls. CMV IgM antibodies were negative in all the nurses studied. Only one person in the control group was positive for the CMV IgM antibodies (Table 2).

**Table 2:** Prevalence of IgG antibodies to CMV and EBV in study subgroups and control group

	Nurses pediatrics (n = 30)	Nurses transplantology (n = 30)	Nurses primary health care (n = 30)	Control group (n = 30)	*P*‐value
CMV IgG					NS^a^, *
Seropositive [n (%)]	27 (90)	24 (80)	28 (93.33)	26 (86.70)	
Seronegative [n (%)]	3 (10)	6 (20)	2 (6.70)	4 (13.33)	
EBV VCA IgG					
Seropositive [n (%)]	30 (100)	30 (100)	30 (100)	30 (100)	
Seronegative [n (%)]	0	0	0	0	

Abbreviation: NS, not significantly.

* Chi‐2 = 2667, *P* = .45

A statistically significant difference was found in terms of age in the total (n = 90) between nurses with a positive CMV IgG (n = 79) and nurses with a negative CMV IgG (n = 15). CMV IgG‐positive nurses were significantly older (*P* = .02), but no statistically significant difference was found between nurse subgroups and the control group which may be related to the small size of the subgroups.

A comparative analysis of the CMV antibodies titer in the IgG (AU/mL) class did not show a statistically significant difference between nurse subgroups and the control group. Moderately strong correlation was found in the pediatric nurses between CMV IgG antibodies levels and age (*r* = 0.41, *P* = .02) and length of work (*r* = 0.54, *P* < .01). The level of CMV IgG in this group increased with age and length of work.

#### 3.1.2 EBV

EBV VCA IgG antibodies were found in all the nurses studied and in all the participants from the control group. EBV VCA IgM antibodies were negative in all nurses. Only one person in the control group was EBV VCA IgM antibody positive. (Table 2).

A comparative analysis of the titer of EBV VCA IgG antibodies did not show statistically significant differences between the study group and the control group. What the analysis did show was moderately strong correlation between the age of the study participants and the level of EBV VCA IgG antibodies in the group of nurses working in pediatrics (*r* = .55 *P* < .01), and a result close to statistical significance among nurses working in primary health care (*r* = 0.34 *P* = .07). Nurses working in pediatrics (and in primary health care also manifested statistically significant positive correlation between the length of work and the EBV VCA IgG level, (*r* = .49 *P* = .01) and (*r* = .37 *P* = .05), respectively. The more advanced the age and the longer the length of work, the higher the level of the titer of VCA IgG antibodies in the groups.

#### 3.1.3 CMV/EBV PCR

CMV DNA was detected in two nurses. The presence of EBV DNA was confirmed in five nurses (Table 3). A statistical analysis did not reveal correlation between people with positive and negative EBV PCR results between the groups (Table 3). Neither CMV DNA nor EBV‐DNA were detected in any of the 30 women from the control group.

**Table 3:** Distribution of the positive and negative PCR CMV/EBV results in the study

	Nurses pediatrics (n = 30)	Nurses transplantology (n = 30)	Nurses primary health care (n = 30)	Control group (n = 30)	*P*‐value
CMV PCR					
Positive [n (%)]	1 (3.33)	1 (3.33)	0	0	
Negative [n (%)]	29 (96.70)	29 (96.70)	30 (100)	30 (100)	
EBV PCR					
Positive [n (%)]	3 (10)	1 (3.33)	1 (3.33)	0	NS^b^, *
Negative [n (%)]	27 (90)	29 (96.70)	29 (96.70)	30 (100)	

Abbreviation: NS, not significantly.

* Chi‐2 = 3.695, *P* = .27

## 4 DISCUSSION

The study showed CMV IgG seroprevalence of nearly 88% in the studied group nurses as a whole. The percentage of CMV IgG antibodies in nurses in Poland is much higher than in other countries, for instance, in Germany (49.50%)^7^ or France (60%).^8^ However, differences in the organization of healthcare units in Poland and abroad do not allow for making a relevant comparison. It can be supposed that they can have their roots in the difference in the scope of duties of nurses in Poland and abroad where nursing care over patients is provided by nursing assistants, whereas in Poland by nurses. On the other hand, the prevalence of CMV IgG antibodies among women in other countries was similar to that in Poland.^9^ Like in Poland, CMV IgG seroprevalence among the nurses studied, though high, is not higher than among the population of reproductive‐age women.^9^, ^10^

EBV VCA IgG antibodies were found in all the nurses covered by the study as well as in all the participants in the control group. These results are consistent with the results of studies of the EBV seroprevalence in different populations worldwide.^11^, ^12^ Unfortunately, scientific literature does not provide data on the prevalence of EBV VCA IgG antibodies among the professional group of nurses in other countries which would allow to assess and compare the obtained research findings.

The study did not reveal statistically significant differences between the compared groups of nurses and also the control group in terms of CMV IgG seroprevalence. The results seem to indicate that the performance of the profession of the nurse in a pediatric or transplantology ward or in a primary healthcare unit does not involve a higher risk of a transmission of CMV infection than the performance of nonhealthcare‐related professions. On the one hand, these findings can seem surprising, in particular since both pediatric and transplantology wards belong to areas with a high prevalence of CMV and EBV in the patient population.^13^, ^14^ However, 5‐year prospective studies on the transmission of the CMV carried out by Balfour et al^15^ show that seroconversion in nurses in transplantology and dialysis wards was not linked to CMV infection in their patients. Thus, the results obtained in this study are consistent with some studies published in the 80s and 90s in which Lipscomb et al^16^ confirmed absence of association between seropositivity "high‐risk" patients, such as infants and immunosuppressed individuals. Dworsky et al^17^ and Balcarek et al^18^ also indicated that people working in pediatric wards are not significantly more exposed to CMV infection than people who do not professionally deal with children. Similarly, in 2016, Stranzinger et al^7^ did not observe an increased risk of CMV infection in nurses when compared with other professional groups employed in the same pediatric hospital. In the same years (80s and 90s), Haneberg^19^ and Friedman et al^20^ presented in their studies a reverse situation and showed that the incidence of CMV antibodies among women working in pediatric hospitals was higher among women having closer contact with hospitalized children than among workers having less or no contact with children. Similar findings were reported by Sobaszek et al^21^ and Lepage et al^8^ who observed that the prevalence of CMV IgG antibodies is more common not among pediatric nurses but among nursing assistants working in these wards. The position of a nursing assistant involves more frequent and longer contact with the patient and the patient's biological material resulting from the provision of nursing care. On the other hand, the work of the nurse often involves purely technical procedures connected with, for instance, taking blood samples, giving injections and thus with procedures requiring strict hand hygiene. And it is the observance of proper hand washing and standard care measures that seem to be a key security element in the prevention of CMV and EBV.^6^

Age is one of the factors linked to the high prevalence of both CMV and EBV antibodies detection.^2^, ^22^ This study confirmed that nurses with IgG CMV‐positive antibodies were actually older. In addition, significant positive correlation was noted between the age, the length of work, and the CMV IgG antibodies titer. The level of IgG antibodies increased with the age and the length of work of the nurses studied. This correlation was found only in pediatric nurses. In the case of the EBV, VCA IgG antibody titers increased with the age and the length of work of the nurses studied among both pediatric and primary healthcare nurses. It should be said that against other groups studied, the diversity of the EBV VCA IgG titers level results in the group of nurses working in primary health care is clearly higher. This situation did not apply to IgG CMV. Hence the observed correlation in this group could be visible even in the case of such small age differences. The correlation might be linked to the much more frequent reactivation of a latent CMV/EBV infection among pediatric nurses. Although the mechanisms responsible for the reactivation of viruses are not fully known, it is believed that it is the deficit of immune response, inflammatory processes and stress that can play a key role in the reactivation of the infection. The working environment of the nurse is linked to numerous stress‐generating factors. Aside from inadequate remuneration, work overload, understaffing, the nurse has to cope with high responsibility for the work performed and the pressure of being in permanent readiness.^23^ There is also a significant difference between the work of the pediatric nurse and a nurse working with adults. A lot of procedures require the presence of more than one nurse. This can often add to increasing both the pressure of time and the intensity of work. What is required is particular attention, exceptional precision and accuracy of work. Consequently, nurses from pediatric wards can be exposed to a higher level of stress than nurses working in other wards and a reactivation of the CMV/EBV virus can thus occur in this group more frequently. This can suggest that the age and the existence of a negative influence of the working environment contribute to the growth of the titer of antibodies being an indicator of the reactivation of infection. Unfortunately, the literature available does not provide data as regards correlation between the level of CMV IgG/EBV VCA IgG antibodies in nurses and work in different areas of health care.

Neither does the literature available provide reports on molecular biology‐based studies concerning the incidence of CMV and EBV infection among the professional group of nurses. Yet, Demmler et al,^24^ Adler et al^25^ and Morgan et al^26^ applied PCR‐method‐based molecular studies to assess the CMV transmission from pediatric clinic patients to nursing staff. Their studies did not reveal a link between CMV infection in patients and CMV infection among the nursing staff of the clinics involved. In this study, positive CMV DNA results were reported in two cases (2.22%). In the case of the EBV, the genetic material was detected in five nurses (5.55%) from the study group. The presence of the CMV/EBV was not detected in any member of the control group. It should be considered whether the percentage of the positive PCR CMV/EBV findings in the group of nurses studied results from the fact that the work of the nurse entails a much higher exposure to these pathogens than in people not being professionally involved in patient care. The professional tasks performed by the nurse create potentially higher possibilities of infection. The more so since, in this study, the number of people with positive DNA CMV/EBV was found to be more common among the pediatric nurses studied.

In conclusion it should be said that further nurses‐focused studies seem absolutely necessary, the more so since studies aimed at determining the prevalence of CMV/EBV antibodies in this professional group in Poland are still missing. The key element of the assessment of the professional risk should be the provision of safe and hygienic working conditions. Obviously, this subject, and in particular, possible health consequences of long‐term exposure to contact with both CMV^27^, ^28^ and EBV^29^, ^30^ should be given more attention.

## 5 LIMITATIONS

This study has some limitations. The study groups were relatively small, selected in a nonrandomized way. This is the weakness of the study as it does not guarantee the representative character of the group and thus makes generalization of the results impossible. Moreover, the assessment did not include other variables which might have an influence on the development of the infection. What the study indicates is merely the scale of the spread of the CMV/EBV infections. It says nothing about, for instance, the immunity of the people studied.

## DISCLOSURE

*Approval of the research protocol*: The study was approved by the Bioethics Committee of the Medical University of Warsaw (No. KB/140/2014). *Informed consent*: All participants signed informed consent for the original trial. *Registry and the Registration No. of the study/Trial*: N/A. *Animal Studies:* N/A. *Conflict of Interest*: The authors declare that they have no conflicts of interest.

## AUTHOR CONTRIBUTIONS

PZ involved in research project, study group and control group selection, collect blood samples, interpretation of the results of the statistical analysis, and writing a manuscript. BCzP involved in the concept and design of the study. AWT performed laboratory analyses of the samples. PZ, BCzP, and AWT involved in interpretation of the results of laboratory tests. All authors read and approved the final manuscript.
